# Early Renal Involvement in Cats with Natural Feline Morbillivirus Infection

**DOI:** 10.3390/ani10050828

**Published:** 2020-05-10

**Authors:** Paolo Emidio Crisi, Francesco Dondi, Eliana De Luca, Morena Di Tommaso, Kateryna Vasylyeva, Enea Ferlizza, Giovanni Savini, Alessia Luciani, Daniela Malatesta, Alessio Lorusso, Andrea Boari

**Affiliations:** 1 Faculty of Veterinary Medicine, Veterinary University Hospital, University of Teramo, Località Piano d’Accio, 64100 Teramo, Italy; edeluca@unite.it (E.D.L.); mditommaso@unite.it (M.D.T.); aluciani@unite.it (A.L.); aboari@unite.it (A.B.); 2 Department of Veterinary Medical Sciences, Alma Mater Studiorum-University of Bologna, Via Tolara di Sopra 50, 40064 Ozzano dell’Emilia (BO), Italy; f.dondi@unibo.it (F.D); kateryna.vasylyeva2@unibo.it (K.V.); enea.ferlizza2@unibo.it (E.F.); 3 Istituto Zooprofilattico Sperimentale dell’Abruzzo e del Molise (IZSAM), Campo Boario, 64100 Teramo, Italy; g.savini@izs.it (G.S.); d.malatesta@izs.it (D.M.); a.lorusso@izs.it (A.L.)

**Keywords:** feline morbillivirus, urine protein electrophoresis, urine chemistry, chronic kidney disease

## Abstract

**Simple Summary:**

Feline morbillivirus is a newly discovered paramyxovirus infecting domestic cats. Its pathogenetic role in domestic cats is still debated, however some evidences suggest a potential involvement of this novel feline virus in the pathogenesis of feline chronic kidney disease. In this study, clinical data of cats infected by morbillivirus were retrospectively reviewed and compared with data obtained from healthy cats and cats affected by chronic kidney disease. The results of the present study suggest that this infection can be associated with the presence of a sub-clinical kidney damage and with different grades of renal dysfunction in cats younger than those typically affected by chronic kidney disease.

**Abstract:**

Feline morbillivirus (FeMV) is a newly discovered paramyxovirus infecting domestic cats and its role in the pathogenesis of feline chronic kidney disease (CKD) has been suggested, however not confirmed. The primary aim of the study was to evaluate the renal damage associated with FeMV infection in cats. In this retrospective study, clinical and clinicopathological data were compared among 14 FeMV naturally infected, 21 CKD and 22 healthy cats. FeMV positive cats had serum chemistry analytes and main urine chemistry results similar to the healthy subjects. FeMV positive cats had significantly decreased urine specific gravity (median 1054, range 1022–1065) and urine creatinine (median 227.23 mg/dL, range 83.02–489.75) when compared with healthy cats (median 1067, range 1040–1080, *p* < 0.001; median 406.50 mg/dL, range 195.32–575.58; *p* < 0.001, respectively). Urine protein:creatinine ratio (UPC) results of FeMV and CKD were not different (median 0.20, range 0.08–1.03; median 0.23, range 0.10–0.80, respectively), however UPC results were significantly increased in both groups, if compared with healthy cats (median 0.1, range 0.04–0.250, *p* < 0.01). Based on clinical data, serum creatinine concentration, urine specific gravity and UPC results, CKD was suspected by clinicians in 3/14 FeMV cats. Urine protein sodium-dodecyl-sulfate-polyacrylamide gel electrophoresis (SDS-PAGE) in 10/13 (77%) FeMV cats indicated a tubular pattern, with a decrease of uromodulin and an increase in the number and intensity of low molecular weight proteins. FeMV infection can be associated with different grades of renal dysfunction ranging from mild tubular proteinuria with less concentrated urine to azotemia in cats younger than those typically affected by CKD.

## 1. Introduction

Feline morbillivirus (FeMV), family *Paramyxoviridae*, was first discovered and isolated in stray and domestic cats in Hong Kong in 2009 [1]. Later, the presence of FeMV was reported in cats from Japan [2,3], Italy [4,5,6], Germany [7], USA [8], Turkey [9], Brazil [10] and the United Kingdom [11]. Some of these studies suggested a possible association between FeMV infection and tubulointerstitial nephritis (TIN), which represents the most frequent histopathological finding in feline chronic kidney disease (CKD) [1,2,3,9,12], even though a clear association between FeMV infection and the development of CKD is lacking.

CKD is a common feline disease with a reported prevalence ranging from 1 to 3% in the general population and reaching 30% in geriatric cats [13]. Therefore, an early diagnosis of CKD is needed to prevent further damage and to reduce the progressive decline of renal function. The determination of the glomerular filtration rate (GFR) is still considered the most sensitive index for the evaluation of renal function, but it is impractical for routine clinical use, particularly in cats [14]. At an early stage, histopathological examination of renal tissue can help to identify the presence of kidney disease and to obtain a pathological diagnosis of renal damage, including TIN [15]. However, the renal biopsy is an invasive procedure with some potential adverse effects [16] and in most cases is unable to recognize the etiology of the renal injury [12,15]. Currently, in feline clinical practice, CKD diagnosis and staging are mainly based on serum creatinine concentration (sCr), urine concentrating ability, quantitative proteinuria measured by urine protein:creatinine ratio (UPC) and imaging results [17]. In addition, these variables are late markers and are more useful to detect advanced stages of CKD. Serum symmetric dimethylarginine (SDMA) has been proposed as an early indicator of CKD, as reported by the International Renal Interest Society guidelines (IRIS) [18], although its early diagnostic role needs further evidence [19]. For these reasons, other sensitive and specific biomarkers, measurable in non- or minimally invasive biological samples, are required in clinical practice to identify early renal damage.

Urine represents a readily available source of biomarkers for the evaluation of renal function or damage. In particular, qualitative proteinuria, by means of proteomic techniques, and urinary fractional excretion (FE) of electrolytes have been studied in the evaluation of renal damage in cats and dogs [20,21,22]. Regarding qualitative proteinuria and proteomics, sodium-dodecyl-sulfate-polyacrylamide gel electrophoresis (SDS-PAGE) and two-dimensional gel electrophoresis have been applied in cats, leading to the identification of putative biomarkers of tubulointerstitial damage in feline CKD including uromodulin, cauxin and retinol binding protein (RBP) [21,22,23].

Previously reported studies have suggested a correlation between viral infection and the presence of TIN in affected cats, hypothesizing the involvement of FeMV in the pathogenesis of feline CKD. Notwithstanding, knowledge on the clinical implications and role of FeMV in feline nephrology is still lacking.

Therefore, the aim of the study was to identify and characterize renal disease in cats naturally infected with FeMV using clinical and clinicopathological data including SDS-PAGE and urine chemistry.

## 2. Materials and Methods

This was a retrospective, cross-sectional study, performed between July 2015 and March 2017 at two Veterinary University Hospitals (VUH) (Faculty of Veterinary Medicine, University of Teramo, and Department of Veterinary Medical Science, University of Bologna, Italy). Cats included in the study were divided into three groups: FeMV positive cats, CKD cats and healthy cats. The FeMV group was composed of client-owned cats, from different sites in the Abruzzo and Emilia Romagna regions, naturally infected with FeMV and with evidence of urinary shedding of the viral genome [24]. These cats were diagnosed during a wider epidemiological evaluation regarding the diffusion of FeMV in a cat population [6]. The CKD group was composed of cats with naturally occurring CKD; in particular, cats were diagnosed with CKD based on the presence of consistent history, clinical, laboratory and imaging findings. Included cats must have evidence of abnormal renal size and architecture as determined by abdominal ultrasonography (Logiq S8; GE Healthcare, Solingen, Germany) and either (a) persistent azotemia (serum creatinine concentration [sCr] >1.6 mg/dL), or (b) persistently inadequate urine specific gravity (USG < 1035), both assessed and confirmed over 1-month [17]. Only cats that were re-evaluated on at least two consecutive occasions 2–4 weeks apart were eligible for inclusion in the study. CKD cats were staged according to International Renal Interest Society (IRIS) guidelines for feline CKD staging, and as previously reported [17], as follows: stage 1: sCr < 1.6 mg/dL; stage 2: sCr 1.6–2.8 mg/dL; stage 3: 2.9–5.0 mg/dL; stage 4: > 5.0 mg/dL.

Cats were excluded from the CKD group if they had acute kidney injury (AKI) or decompensated CKD, or if they were affected by non-renal diseases or treated with therapies known to influence USG and proteinuria, regardless of the presence of renal disease itself (e.g., hypercalcemia, diuretics, fluid therapy; pre-renal, post-renal or extra-urinary proteinuria, hyperthyroidism, hypertension non related to CKD, respectively).

Healthy group: during the same study period, client-, student- and hospital staff-owned as well as blood donor cats were enrolled as a control group. These cats underwent to the same evaluation of FeMV and CKD cats and were defined healthy based on history, physical examination including blood pressure measurement, imaging results and blood and urine screening tests. Healthy and CKD cats must result FeMV-negative using the same diagnostic protocol used in FeMV cats.

### 2.1. FeMV Infection Diagnosis and Serological Evaluation

FeMV infection was diagnosed by the demonstration of FeMV RNA in the urine, as described in a previous paper [24].

Immunofluorescence (IF) was also performed to detect antibodies (Ab) to FeMV-N protein in cat sera [6]. The Ab titers of tested cat sera were recorded as the reciprocals of the highest dilutions producing positive staining. Ab titers of less than 1:40 were considered negative.

### 2.2. Clinical and Clinicopathological Evaluation

All cats underwent to a complete physical examination, including blood pressure measurement, and abdominal ultrasound. Body weight and 9-point body condition score (BCS) were recorded. Doppler (Minidop ES-100VX; Hadeco, Kawasaki, Japan) or oscillometric (petMAP graphic; Ramsey Medical, Tampa, FL, USA) systolic blood pressure (SBP) measurements were performed as recommended by IRIS guidelines and as reported by Taylor and colleagues [25]. Cats were considered hypertensive if their SBP was persistently above 160 mmHg.

Blood samples were obtained for all cats using blood vacuum systems (Vacutest Kima; Arzegrande, Italy), after at least a 12-h fasting period. Concurrent urine samples were obtained by spontaneous voiding or cystocentesis. The following analyses were performed: venous blood gas analysis (ABL 800 Flex; Radiometer Medical ApS, Brønshøj-Husum, Denmark) including pH, base excess (BE), anion gap, lactate and ionized calcium (iCa) concentration; complete blood count (CBC) (ADVIA 2120; Siemens Healthcare Diagnostics, Tarrytown, NY, USA) and blood smear examination; serum chemistry profile (OLYMPUS AU 400; Olympus-Beckman Coulter, Brea, CA, USA) including sCr, albumin, total proteins, glucose, alanine transaminase (ALT), aspartate transaminase (AST), alkaline phosphatase (ALP), total bilirubin, gamma(γ)-glutamyltransferase (GGT), total cholesterol, sodium, potassium, magnesium, chloride, total calcium and phosphate. The serum SDMA concentration was also determined from serum stored at −20 °C (IDEXX SDMA; IDEXX Laboratories, Milano, Italy). Feline Immunodeficiency Virus (FIV) antibody and Feline Leukaemia Virus (FeLV) antigen were evaluated with a commercial assay kit following manufacturer instruction (SNAP FIV/FeLV Combo Test; IDEXX Laboratories, Westbrook, ME, USA). Urinalysis including refractometric urine specific gravity (USG) (American Optical, Buffalo, NY, USA), dipstick examination (Combur10Test; Roche Diagnostic, Basel, Switzerland) and microscopic evaluation of the sediment, were performed. In cats with active sediment, bacteriuria or suspected urinary tract infection (UTI), urine culture was performed, when required by attending clinicians. Standardized technique was used for bacterial culture: urine samples were plated onto 5% sheep blood agar and incubated at 37 °C for 48 h.

Samples with severe hematuria (gross hematuria or ≥100 red blood cells at ×400), pyuria or both, as defined previously, were excluded from urine chemistry and SDS-PAGE [26].

Urine chemistry evaluation included urine creatinine (uCr), urine total protein (uTP) and other urinary analytes concentration measurement (urea, uUrea; calcium, uCa; phosphate, uP; sodium, uNa; potassium, uK; magnesium, uMg; chloride, uCl). The uTP:uCr ratio (UPC) was calculated as follows: UPC = uTP (mg/dL)/uCr (mg/dL). Proteinuria and borderline proteinuria were considered when UPC value were above 0.4, and between 0.2 and 0.4, respectively [18].

Fractional excretion of urinary analytes was calculated by the following formula:

FEe = uₑ × sCr/uCr × s_e_ × 100 (based on spot urine sample) (1)

where “e” indicated the analyte, and “u” and “s” indicated urine and serum values, respectively. Urinalysis was performed within 2 h from the collection and then urine supernatants, after centrifugation for 10 min at 1500× *g*, were stored at −80 °C for the further evaluations. Urine chemistry and SDS-PAGE were performed on stored supernatants within 1 month from the collection; in particular, urine proteins were separated using an electrophoretic system (NuPage; Thermo Fisher Scientific; Waltham, MA, USA) on precast 12% polyacrylamide gel in reducing conditions with a MOPS (3–(N–morpholino) propanesulfonic acid) buffer containing sodium-dodecyl-sulfate (Thermo Fisher Scientific; Waltham, MA, USA). Five micrograms of proteins for each sample were loaded. Gels were stained with Coomassie blue staining (PageBlu protein staining solution; Thermo Fisher Scientific; Waltham, MA, USA). After staining, each gel was digitalized by a semiautomated system (ChemiDoc MP; Bio-Rad Laboratories, Hercules, CA, USA) and pherograms were obtained using a commercial software (ImageLab 5.2.1 Software; Bio-Rad Laboratories, Hercules, CA, USA). To avoid manual correction of band, the same optimized default settings (background subtraction disk size 5.00 mm, sensitivity 20.00, size scale 5, noise filter 4, shoulder 1) were applied to each gel and used to detect and count protein bands. Each band was verified by two of the authors (E.F., F.D.). Protein bands were classified as high molecular mass (HMM) proteins when presenting a molecular mass equal or above to albumin, and low molecular mass (LMM) proteins when below to albumin. Electrophoretic patterns were evaluated qualitatively and quantitatively by the enumeration of total, HMM and LMM number of bands.

### 2.3. Renal Histopathology and Immunohistochemistry

When available, kidney tissue samples of deceased cats belonging to the FeMV group were submitted for histopathological and immunohistochemical (IHC) analyses, as reported previously [6,24].

Briefly, all renal tissues, resulted FeMV positive by quantitative polymerase chain reaction (qPCR), were fixed in 10%neutral buffered formalin, embedded in paraffin and cut into 3-μm-thick sections, mounted on positive charged glass slides, dewaxed and rehydrated using standard procedures and stained by Haematoxylin and eosin (HE). For IHC, 3-μm-thick tissue sections were dewaxed and rehydrated and then incubated with 3% hydrogen peroxide diluted in absolute methanol for 30 min to inhibit endogenous peroxidase activity and then rinsed in 0.05 M Tris-buffered saline (TBS), pH 7.6, for 5 min. Antigen retrieval was performed by heat treatment in citrate buffer 0.01 M pH 6.0 at 121 °C for 5 min. To reduce nonspecific binding, slides were incubated with 20% normal swine serum (Dako, Copenhagen, Denmark) diluted in TBS for 20 min, followed by a second incubation with 10% skimmed milk diluted in TBS for 20 min at room temperature. Then tissue sections were incubated overnight in a humidified chamber at 4 °C with the rabbit polyclonal antibody against the N protein of FeMV. After three washing in TBS, tissues sections were incubated with an horseradish peroxidase-labeled polymer conjugated with the secondary anti-rabbit antibody (REAL™Envision detection system peroxidase Dako, Copenhagen, Denmark) for 30 min. Immune reactions were detected by means of a peroxidase technique (REAL™Envision detection system peroxidase Dako, Copenhagen, Denmark) and visualized using 3-3′-diaminobenzi-dine as chromogen. Positive and negative controls were included in each IHC run. In this respect, the specificity of immunolabeling was verified by testing the same IHC protocol on qPCR FeMV negative renal sections, with and without inflammatory lesions. In addition, all samples were tested with an irrelevant antibody directed against an unrelated antigen (anti-Von Willebrand factor rabbit polyclonal Ab 1:800, DakoCytomation, Denmark).

### 2.4. Statistical Analysis

Statistical analysis was performed using a statistical software (MedCalc version 17.6; MedCalc Software Ltd, Ostend, Belgium). All data were evaluated using standard descriptive statistics and reported as mean ± standard deviation (SD) or as median and range (minimum–maximum), based on their distribution. Normality was checked graphically or using the D’Agostino Pearson test. Variables collected were compared among the three groups using the ANOVA or a Kruskall–Wallis test and post-hoc tests (Student–Newman–Keuls test or Dunn test). Categorical variables were analyzed using the χ² test. A *p*-value < 0.05 was considered significant.

## 3. Results

A total number of 57 cats were included in the study. Of these, 14/57 cats were FeMV PCR positive and included in the FeMV group, 21/57 in the CKD group and 22/57 were enrolled as healthy control group. Cats belonging to CKD and Healthy groups were FeMV PCR and IF negative.

In the FeMV group, 8/14 (57%) infected cats were neutered males and 6/14 (43%) were females (5 spayed). The median age was 35 months (range 14–101), and all of the animals were European domestic cats. In PCR positive cats, the median Cycle threshold value was 33.5 and ranged between 32 and 39. Within this group, sera of 12/14 (86%) cats underwent IF in order to detect antibodies against specific FeMV–N protein. Median IF titer was 1:2560 and ranged between 1:160 and 1:10,240; one cat was negative to serology.

In the CKD group, 14/21 (67%) cats were males (12 neutered), and 7/21 (33%) were spayed females. The CKD cats were significantly older than FeMV and healthy cats with a median age of 174 months (range 40–205; *p* < 0.01). Breeds included in the CKD group were European domestic cats (18/21), Persian (1/21), Chartreaux (1/21) and Siamese (1/21). Of the CKD cases, 2/21 (10%) were classified as IRIS stage 1, 7/21 (33%) stage 2, 8/21 (38%) stage 3 and 4/21 (19%) stage 4.

The healthy control group included 11/22 (50%) males (8 neutered) and 11/22 (50%) females (9 spayed) cats with a median age of 55 months (range 12–109). Breeds represented in this group included European domestic cats (15/22), Main Coon (4/22), Sphynx (1/22), Chartreaux (1/22) and Devon Rex (1/22). FeMV positive cats and healthy subjects had similar age (*p* = 0.15), while cats of both groups were significantly younger than CKD cats (*p* < 0.001). There was no significant difference in sex among the three groups.

Body weight and BCS were not different between FeMV and CKD cats, however they were significantly lower if compared with healthy ones (*p* = 0.005 and *p* = 0.001, respectively) (Table 1).

Among the FeMV positive cats, 2 tested positive for FeLV while no cats tested positive for FIV.

The main clinical observations and imaging features of cats belonging to the healthy, FeMV and CKD groups are summarized in Table 1.

### 3.1. Hematology, Chemistry and Urinalysis Results

The results of CBC and serum chemistry, and their comparison between the three groups are reported in Table 2. 

Serum creatinine was above the study cut-off values in 1/14 (7%) FeMV and 19/21 (90%) CKD cats and USG was persistently below to 1035 in 1/14 (7%) FeMV and 19/21 (90%) CKD cats; the both abnormalities were simultaneously present in none of the FeMV and in 18/21 (86%) CKD cats, respectively. Based on these results, CKD was suspected by clinicians in 2/14 (14%) FeMV cats (IRIS stage 1, n = 1; IRIS stage 2, n = 1). SDMA results were available for 14/14 FeMV and 18/21 CKD cats. None of the FeMV and 12/18 CKD cats had SDMA above the IRIS CKD suggested threshold (≥18 μg/dL) [18]. FeMV cats had significantly lower sCr, urea, SDMA, total and ionized calcium concentrations as compared with CKD cats. Serum phosphate of FeMV cats was significantly higher than in healthy cats but no difference was detected by the comparison with the CKD group. Albumin concentration and albumin:globulin ratio (A:G) of FeMV and CKD groups were not different, however both were significantly lower as compared with healthy cats. In FeMV cats, total cholesterol and total bilirubin concentrations were significantly lower than in CKD and healthy cats. A reduced activity of ALP was observed in both FeMV and CKD groups as compared with healthy cats. The FeMV cats had significantly higher serum potassium concentrations than healthy and CKD groups, and higher chloride concentration than healthy cats. There were no differences in serum sodium and magnesium concentration among the three groups.

No differences were observed for other serum chemistry analytes among the three groups.

### 3.2. Quantitative and Qualitative Urine Analysis

Urine culture was performed in 13/14 (93%) FeMV and 2/21 (9%) CKD, and yielded negative results in all these cats. One cat included in the FeMV group was excluded from urine chemistry (including UPC evaluation) and SDS-PAGE due to the presence of relevant hematuria, as defined above. Pyuria was never detected in the cats included in the study.

When compared with the healthy ones, FeMV cats had significantly decreased USG, uCr and uUrea and significantly increased UPC. Conversely, FeMV cats had significantly increased USG, uCr and uUrea when compared with the CKD ones (Table 3). UPC values were not different between these two groups. Proteinuria, as previously defined, was detected in 1/13 (8%) FeMV and 6/21 (29%) CKD cats, respectively. Frequency of proteinuria was not significantly different between CKD and FeMV cats (*p* = 0.149).

FE of sodium, potassium, calcium, phosphorous, chloride and magnesium of the FeMV group were not different from the healthy group but were significantly lower than those of the CKD group (Table 3).

**Figure 1:**
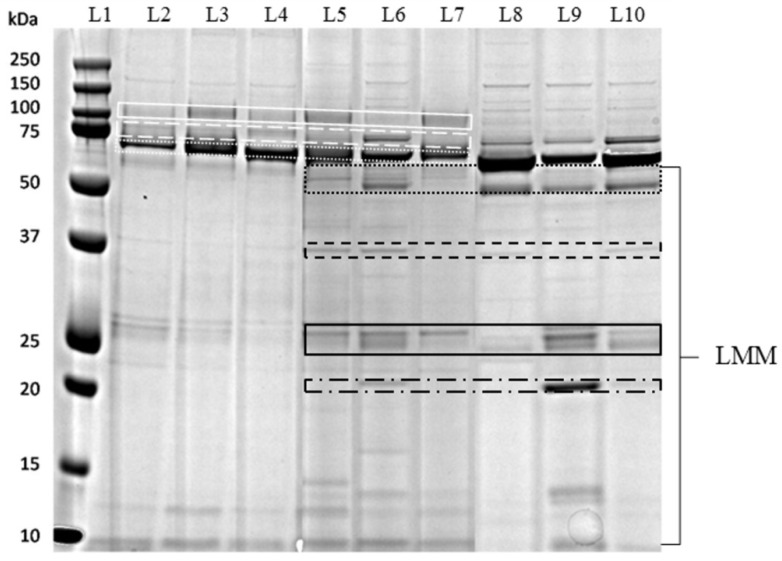
Representative sodium-dodecyl-sulfate-polyacrylamide gel electrophoresis (SDS-PAGE) of cat urine samples: lane 1 (L1), molecular mass marker; lanes 2–4 (L2–L4), urine samples from healthy cats; lanes 5–7 (L5–L7), urine samples from FeMV cats; lanes 8–10 (L8–L10), urine samples from CKD cats. White continuous line indicates uromodulin (100-92 kDa); white dashed line indicates transferrin (76 kDa); white dotted line indicates cauxin and albumin (67-62 kDa); black dotted and black continuous lines indicate immunoglobulin heavy (54 kDa) and light (26 kDa) chains, respectively; black dashed line indicates haptoglobin (36 kDa); black dotted and dashed line indicates retinol binding protein (20 kDa). LMM, low molecular mass.

**Figure 2:**
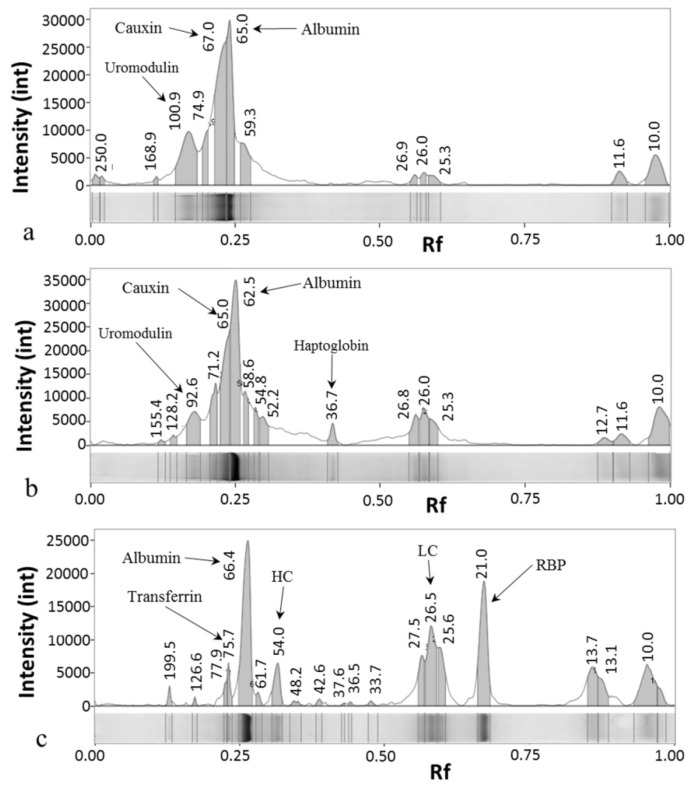
Representative pherograms of cat urine samples: (**a**) Healthy cat (same sample of lane 3 Figure 1); (**b**) FeMV cat (same sample of lane 5 Figure 1); (**c**) CKD cat (same sample of lane 9 Figure 1). The number above each peak correspond to the Molecular Mass of the band in kD. Arrows indicates the peaks corresponding to the bands of uromodulin (100-92 kDa), cauxin (67-65 kDa), albumin (65-62 kDa), transferrin (76 kDa), immunoglobulin heavy chains (HC, 54 kDa) and light chains (LC, 26 kDa), haptoglogin (36 kDa) and retinol binding protein (RBP, 20 kDa). Int, pixel intensity; Rf, ratio of the front.

SDS-PAGE of urine samples was conducted for each group (Figure 1) and the pherograms acquired (Figure 2). In healthy cats, a typical electrophoretic profile was found, common to all the examined samples. This pattern was characterized by the presence of HMM proteins, including uromodulin (94 kDa), transferrin (73 kDa), cauxin (65 kDa) and albumin (62 kDa), and a limited number of LMM proteins. In most samples, the bands of cauxin and albumin were distinguishable, though not quantifiable separately.

In CKD cats, a decrease or disappearance of uromodulin and cauxin was accompanied by an increase appearance of albumin, transferrin and LMM proteins as immunoglobulin light (26 kDa) and heavy chains (54 kDa) and haptoglobin (36 kDa); in most samples the presence of the RBP (20 kDa) was recorded.

FeMV positive cats presented variable profiles, which were in part similar to CKD profiles (Figure 2). Uromodulin (92 kDa) and cauxin (65 kDa) were still present, although in lesser amounts than in healthy cats. The pattern was characterized by an increased number of LMM proteins and few samples presented the RBP band. This profile was detected in 10/13 (77%) FeMV-positive cats. The HMM, LMM and total number of bands detected in the study groups are reported in Table 3. CKD and FeMV cats had an increased number of LMM bands if compared with the healthy ones (*p* = 0.024); HMM bands were significantly decreased in CKD cats if compared to the others (*p* = 0.049). Moreover, FeMV cats had a significantly increased total number of bands if compared with the healthy ones (*p* = 0.016).

### 3.3. Renal Histopathology

**Figure 3:**
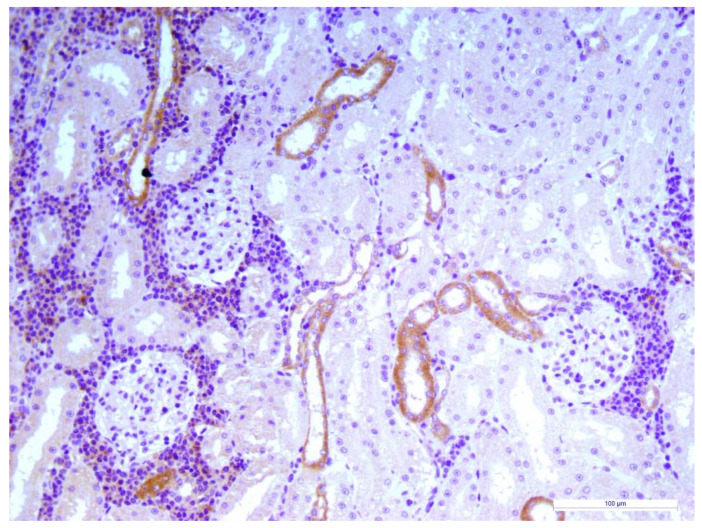
Cat, Kidney, Immunohistochemestry for FeMV N protein. Severe tubulointerstitial nephritis (TIN) with mononuclear cells infiltration. Epithelial cells of some cortical distal tubules showed diffuse and granular immunoreactivity for viral antigen. Mayer’s hematoxylin counterstain, final magnification × 200. Scale bar: 100 μm.

Three FeMV positive cats deceased within two months from the clinical visit because hit by car and renal histopathology showed moderate to severe chronic TIN. In all of the cases, immunohistochemical staining revealed FeMV–N positive cells mostly involving the cortical kidney tubules with an extensive inflammatory infiltrate surrounding the FeMV–N positive cells (Figure 3)

## 4. Discussion

The present study aimed to identify and characterize renal dysfunction in cats infected by FeMV using non-invasive methods. To achieve these goals, clinical and clinicopathological findings, including urinalysis and urine chemistry results, of a population of cats naturally infected with FeMV were retrospectively compared with those obtained from clinically healthy and CKD cats. Since urine SDS-PAGE represents a highly sensitive way to evaluate renal damage in cats, as reported in previous studies [21,23], qualitative proteinuria was included in the assessment of these animals. The retrospective nature of the study and the inclusion of cats affected by spontaneous disease in different stages may not allow to prove a clear cause-effect relationship between FeMV infection and development of renal damage in FeMV infected cats. Nonetheless, in contrast with a previous study that not detected a clear association between urinary excretion or past exposure to FeMV and azotemic CKD [11], the findings of our study suggest that FeMV infected cats have some degree of renal damage while they are shedding virus in their urine.

To date, knowledge on epidemiology of FeMV infection in cats is lacking, and sex, age or breed predisposition is still unknown. In the current study, adult (i.e., >1-year-old) European domestic cats were over-represented in the FeMV group while no differences were highlighted regarding sex. CKD is the most common cause of death in cats, affects 30%–40% of older cats [17] and aging is an important factor implicated in the initiation of the disease [27]. Interestingly, in this study, healthy cats and FeMV cats had similar age and FeMV cats were significantly younger than CKD cats, suggesting that the renal involvement, observed in the cohort of FeMV infected cats, might not be related to the aging process.

The majority of FeMV infected cats had hematological and biochemical results within the reference interval. However, slight differences were observed when they were compared with the healthy cats. In particular, CBC in FeMV positive cats showed lower hematocrit (Hct) values, and higher platelet and white blood cell (WBC) counts. These findings, in association with FeMV infection, are non-specific and potentially related to inflammation experienced by the FeMV cats, although not necessarily related to FeMV infection [28,29].

Creatinine and, most recently, SDMA are useful serum markers of decreased kidney function in cats and dogs [30]. In our study, 13/14 (93%) and 14/14 (100%) FeMV-positive cats had sCr and SDMA concentration, respectively, below the suggested IRIS CKD staging cut-off values for stage 2. Nevertheless, based on the criteria used in our study for the diagnosis of CKD and on the follow-up evaluation, CKD IRIS stage 1 and IRIS stage 2 were suspected in 2/14 FeMV cats. Furthermore, CKD IRIS stage 1 was a consistent diagnosis, suspected by clinicians, in an additional FeMV-positive cat presenting proteinuria (UPC > 0.4) that remained persistently proteinuric during the follow-up period (data not shown). These findings suggest that FeMV might be associated with CKD cases also in cats younger than the senior ones.

When compared with CKD, FeMV cats had a significantly lower sCr concentration. Although lower than the healthy ones, FeMV cats had similar body weight and BCS, if compared with CKD, and for this reason a poorer body condition in FeMV cats could not be considered as a possible explanation of creatinine underestimation. Hyperfiltration experienced by FeMV cats could represent another hypothesis raised to explain this finding; however, it remains difficult to be demonstrated without the evaluation of a measured GFR, and should be confirmed in further studies.

In the present study, although 13/14 (93%) FeMV cats had appropriately concentrated urine (i.e., USG >1035) upon admission and in the follow up period (data not shown), USG results were significantly lower if compared with the healthy group. This finding, together with the lower values of uCr, may suggest an early renal dysfunction in cats infected by FeMV characterized by an impaired ability to concentrate urine despite a normal urine handling of electrolytes, as suggested by the values of FE of electrolytes. Nevertheless, comparing USG in the appropriately elevated range could not be clinically relevant and additional studies using more accurate indicators of renal concentrating ability should be performed to confirm these results. 

The UPC values of FeMV and CKD cats were similar, the frequency of proteinuria was not different between these groups and, furthermore, 6/13 (46%) FeMV positive cats were proteinuric or borderline proteinuric, suggesting a potential kidney damage. Indeed, Jepson and colleagues [20] have identified UPC and USG, among others, as early predictors for the onset of CKD in 118 cats. These findings seemed to be further corroborated when SDS-PAGE results were combined with quantitative proteinuria. SDS-PAGE is helpful for the early diagnosis of renal damage or dysfunction in cats and dogs, and can aid to distinguish between glomerular and tubular proteinuria [21,26,31]. Healthy cats included in this study had a typical pattern characterized by the constant presence of uromodulin and albumin or cauxin bands. Uromodulin and cauxin are known markers of tubular damage and their decrease was already demonstrated in cats affected by kidney disease [21,23,26]. In the CKD group, a lack or decrease of uromodulin and cauxin and an increase of albumin and transferrin were observed. Moreover, the presence of a significantly higher mean number of bands with an LMM compared with healthy cats confirmed the prevalent tubular involvement that characterize CKD in cats, as reported previously [21]. In cats infected by FeMV, SDS-PAGE showed differences when compared with the healthy cats. In particular, FeMV cats had frequently a tubular pattern characterized by an increase in the number and intensity of LMM proteins (not different to CKD cats) and a decrease of uromodulin. Moreover, the appearance of RBP, another known marker of tubular damage [32], in urines of CKD cats and in some samples of FeMV cats, let us confirm the renal involvement also in FeMV cats, although less severe than in the CKD ones.

All tested cats, except one, had detectable antibodies against specific FeMV-N protein. As previously suggested, it is important to interpret with caution serological results. Moreover, combining the results of RT-PCR with serology could be useful to understand the length of exposure in infected cats [33]. Indeed, the seronegativity herein observed in a PCR-positive cat may suggests an acute infection that could have been confirmed by serology only using a convalescent titer in this case.

The three FeMV cats that underwent kidney histopathology revealed tubulointerstitial changes. These lesions are common in cats regardless the presence of renal disease [34] thus, it is hard to interpret and discuss the clinical implication of tubular lesions in positive cats. However, several studies reported a higher incidence of TIN in cats with a FeMV infection compared with negative cats [1,3,9]. In particular, in a recent study, the detection of FeMV antigens was significantly associated with some morphological abnormalities in feline kidney tissues, particularly those in renal tubular and interstitial areas [35].

This study had some limitations, first of all its retrospective nature that makes difficult to establish a clear association of FeMV-positivity with changes in renal function or CKD and that shall be overcome in further studies using experimental infection models. The limited cohort size and the fact that the study groups were not age-matched could be a source of bias in the statistical comparison for some variables. Additionally, despite the use of same instrumentations, analytical methods, calibration and quality control material in the two institutions that performed the clinicopathological evaluation, a potential degree of variation between the laboratories results cannot be completely excluded. Moreover, even though FeMV was detected in cats using a specific molecular assay targeting the viral genome, it is difficult to assess whether infectious virus was still present at the time of urine collection. In this regard, indeed, we were not able to provide clear indications regarding the phase of the infection. To circumvent this issue, an experimental study is therefore highly recommended. Furthermore, the potential role of comorbidities on clinical and clinicopathological findings (e.g., body weight and BCS) in FeMV cats should be taken into account when interpreting our results. However, no concomitant diseases were clearly diagnosed by clinicians at the time of inclusion in the study with the exception of FeLV infection in two cases. Retroviral infections are known to be associated with clinicopathological abnormalities including proteinuria in cats and could have partially biased our results. Nevertheless, the two FeLV-positive cats were non-proteinuric, had no significant laboratory abnormalities and presented a lower number of bands in their urines at qualitative proteinuria evaluation if compared with the other FeMV cats (data not shown). For these reasons, these cats were not excluded from the study. Finally, histopathology and immunohistochemistry would have been necessary in order to characterize the FeMV-associated kidney damage, however, in this study, were available only in three cases.

## 5. Conclusions

Cats affected by FeMV had less concentrated urines if compared with healthy cats; furthermore, they were proteinuric or borderline proteinuric in half of the cases. These findings, associated with the results of urine electrophoresis, suggest a tubular damage or dysfunction in FeMV positive cats, and most of these cases could be classified as CKD IRIS stage 1.

FeMV infection could represent a potential renal insult leading to an early renal dysfunction in the infected cats included in the present study; however, a clear pathogenetic role of the FeMV cannot be determined in a spontaneous disease setting due to other potential confounders.

Finally, further prospective studies combining long-term patient follow-up, renal pathology and urine tests are warranted to obtain a better characterization of potential FeMV-associated renal damage in the clinical setting.

## Figures and Tables

**Table 1:** Main complaints, physical examination and ultrasound findings and retroviral status of healthy cats (n = 22) and cats belonging to the FeMV (n = 14) and the CKD (n = 21) groups.

Variable	Healthy (n = 22)	FeMV (n = 14)	CKD (n = 21)	*p* Value
Median body weight (kg)	5.0 (2.6–7.6)	3.8 (3.0–6.0)	4.0 (2.1–7.4)	0.005 *
Median BCS	6 (5–8)	5 (2–7)	5 (3–6)	0.001 *
Poor coat conditions n (%)	–	8 (57%)	4 (19%)	0.003
Gingivostomatitis n (%)	–	4 (29%)	8 (38%)	0.721
Diarrhea n (%)	–	3 (21%)	2 (9%)	0.369
Hyporexia/Anorexia n (%)	–	2 (14%)	12 (57%)	0.016
FeLV n (%)	–	2 (14%)	0	0.153
FIV n (%)	–	0	0	1.000
Weight loss n (%)	–	1 (7%)	11 (52%)	0.009
Vomiting n (%)	–	1 (7%)	6 (29%)	0.203
Pale mucous n (%)	–	1 (7%)	6 (29%)	0.203
Lethargy n (%)	–	1 (7%)	3 (14%)	0.635
Enlarged abdomen n (%)	–	1 (7%)	0	0.400
Dehydration n (%)	–	1 (7%)	7 (33%)	0.108
PU/PD n (%)	–	0	10 (48%)	0.002
Heart murmur n (%)	–	0	3 (14%)	0.259
Constipation n (%)	–	0	2 (9%)	0.506
Abnormal renal palpation n (%)	–	1 (7%)	7 (33%)	0.108
Hypertension (SBP > 160 mmHg) n (%)	–	0	9 (43%)	0.005
Ultrasound abnormalities n (%)	3 (14%)	2 (14%)	21 (100%)	<0.001
Hyperechoic cortex	3 (14%)	2 (7%)	21 (100%)	<0.001
Decreased corticomedullar distinction	–	0	13 (62%)	<0.001
Small kidneys	–	0	5 (24%)	0.063
Irregular kidney shape	–	0	10 (48%)	0.002

Data were reported as median and range (in brackets) or as number of cases and percentage (%). FeMV = Feline morbillivirus; CKD = chronic kidney disease; BCS = body condition score; FeLV = Feline Leukaemia Virus; FIV = Feline Immunodeficiency Virus; PU/PD = polyuria and polydipsia; SBP = Systolic Blood Pressure; * Statistically significant difference in Healthy compared with CKD and FeMV cats.

**Table 2:** Complete blood count and serum chemistry values of cats belonging to the three study groups: Healthy (n = 22), FeMV (n = 14) and CKD (n = 21).

Variable	RI	Healthy (n = 22)	FeMV (n = 14)	CKD (n = 21)	*p* Value
Hct %	32–48	38.3 (33.3–48.7)	32.0 (19.9–41.4)	27.0 (17.7–40.1)	<0.001 *
WBCs cells/mm³	4800–14,900	8600 (4980–18,830)	16,345 (7990–23,340)	9660 (5480–27,970)	<0.001 ^¶^
Neutrophils cells/mm³	1600–10,000	4710 (1570–13,520)	9320 (4080–16,870)	6270 (2740–24,790)	<0.001 *
Lymphocytes cells/mm³	900–5600	3050 (1150–4610)	4530 (1660–9700)	1760 (800– 7770)	<0.001 *
Monocytes cells/mm³	0–650	135.0 (50.0–490.0)	470.0 (0.0–920.0)	240.0 (70.0–870.0)	<0.001 *
Eosinophils cells/mm³	60–1470	645 (60–1260)	955 (330–3910)	490 (200–1060)	0.009 ^¶^
Platelets cells/mm³	150,000–500,000	208,000 (45,000–563,000)	362,000 (58,000–615,000)	332,000 (95,000–618,000)	0.033 ^†^
Basophils cells/mm³	0–60	20 (0–40)	20 (10–60)	10 (0–30)	<0.001 ^‡^
Creatinine mg/dL	0.8–1.6	1.46 (0.78–1.90)	0.82 (0.67–2.13)	2.98 (1.52–9.49)	<0.001 *
Creatinine > 1.6 mg/dL, n (%)	–	–	1/14 (7%)	19/21 (90%)	<0.001
Urea mg/dL	30–65	46.48 (31.47–73.01)	45.35 (30.20–63.30)	99.4 (41.51–318.82)	<0.001 ^‡^
SDMA µg/dL	<18	12.5 (7.0–18.0)	10.0 (6.0–15.0)	21.5 (7.0–50.0)	<0.001 ^‡^
SDMA ≥ 18 µg/dL, n (%)	–	–	0/14	12/18 (67%)	<0.001 *
Total calcium mg/dL	8.5–10.5	9.75 (8.80–10.80)	9.20 (8.24–10.38)	10.10 (8.30–11.60)	0.004 ^¶^
Ionized calcium mmol/L	1.19–1.38	1.27 (1.07–1.34)	1.25 (0.80–1.40)	1.32 (1.07–1.50)	0.001 ^‡^
Phosphate mg/dL	2.5–6.2	4.4 (3.0–6.1)	5.43 (3.79–6.50)	4.9 (4.0–12.3)	0.010 ^†^
Sodium mEq/L	145–155	151.0 (147.0–155.0)	149.0 (147.0–154.0)	151.0 (143.0–157.0)	0.299
Chloride mEq/L	110–123	119.0 (113.0–129.0)	120.5 (117.0–125.0)	116.6 (108.4–125.0)	0.022 ^†^
Potassium mEq/L	3.4–5.1	4.2 (3.4–4.9)	4.93 (4.08–5.38)	4.2 (3.1–4.8)	<0.001 ^¶^
Magnesium mg/dL	1.9–2.8	2.30 (2.12–2.48)	2.32 (2.04–2.72)	2.27 (1.80–3.66)	0.952
Total proteins g/dL	6.5–8.8	7.72 (7.06–8.89)	7.87 (7.05–9.12)	7.51 (5.36–9.97)	0.790
Albumin g/dL	2.6–4.0	3.6 (2.0–4.0)	3.1 (2.5–3.7)	3.0 (2.3–3.4)	<0.001 ^†^
A:G	0.52–1.20	0.85 (0.58–1.28)	0.63 (0.37–0.87)	0.67 (0.37–0.90)	0.001 ^†^
Glucose mg/dL	65–148	99.5 (71.0–240.0)	85.0 (62.0–193.0)	106.0 (72.0–148.0)	0.055
AST U/L	9–40	25.5 (16.0–157.0)	23.5 (5.0–65.0)	28.0 (17.0–79.0)	0.419
ALT U/L	20–72	47.5 (33.0–92.0)	55.0 (20.0–117.0)	58.0 (20.0–224.0)	0.309
GGT U/L	0–4	0.1 (0.1–0.6)	0.1 (0.0–1.3)	0.1 (0.1–2.3)	0.590
ALP U/L	20–140	69.0 (22.0–163.0)	26.5 (13.0–120.0)	45.0 (16.0–128.0)	<0.001 *
Total bilirubin mg/dL	0–0.35	0.18 (0.10–0.31)	0.08 (0.05–0.20)	0.19 (0.14–0.32)	<0.001 ^¶^
Total cholesterol mg/dL	59–230	130.5 (45.0–370.0)	86.0 (55.0–142.0)	195.0 (81.0–478.0)	<0.001 *

Data are reported as median and range (in brackets); FeMV = Feline morbillivirus; CKD = chronic kidney disease; RI = reference interval; Hct = haematocrit value; WBCs = white blood cells; SDMA = symmetric dimethylarginine; A:G = albumin:globulin ratio; AST = aspartate transaminase; ALT = alanine transaminase; GGT = gamma(γ)–glutamyltransferase; ALP = alkaline phosphatase; * Statistically significant difference among the three study groups; ^†^ Statistically significant difference in healthy compared with CKD and FeMV cats; ^‡^ Statistically significant difference in CKD compared with healthy and FeMV cats; ^¶^ Statistically significant difference in FeMV compared with CKD and healthy cats.

**Table 3:** Urinalysis and urine chemistry values of cats belonging to the three study groups: Healthy (n = 22), FeMV (n = 14) and CKD (n = 21).

Variable	RI	Healthy (n = 22)	FeMV (n = 14)	CKD (n = 21)	*p* Value
USG	>1035	1067 (1040–1080)	1054 (1022–1060)	1017 (1008–1040)	<0.001 *
USG < 1035, n (%)	–	–	1/14 (7 %)	19/21 (90 %)	<0.001
uCreatinine mg/dL	120–593	406.5 (117.8–575.6)	227.2 (83.0–489.8)	112.7 (48.4–489.6)	<0.001 *
uUrea mg/dL	1500–10700	7597.8 (3650.6–9235.1)	5174.9 (1625.2–6508.3)	2225.8 (967.6–6478.8)	<0.001 *
uTP mg/dL	21–134	41.14 (22.09–151.24)	41.97 (16.44–172.74)	33.42 (5.96–337.90)	0.244
UPC	0.01–0.4	0.1 (0.04–0.25)	0.20 (0.08–1.03)	0.23 (0.10–0.80)	<0.001 ^†^
UPC 0.2–0.4, n (%)	–	–	5/13 (38%)	9/21 (43%)	0.803
UPC > 0.4, n (%)	–	–	1/13 (8%)	6/21 (29%)	0.149
uGlucose mg/dL	4.0–14.0	8.0 (5.0–16.1)	9.9 (3.3–139.0)	2 (0–1408.0)	<0.001 ^‡^
uNa mEq/L	49.0–375.0	210.40 (20.40–296.0)	83.10 (16.20–217.10)	78.80 (13.10–298.20)	<0.001 ^†^
uNa/uCrea	0.15–1.0	0.51 (0.10–0.72)	0.31 (0.07–1.23)	0.76 (0.11–1.20)	0.040 ^‡^
FE Na%	0–0.97	0.49 (0.08–0.88)	0.19 (0.04–0.78)	1.13 (0.21–6.11)	<0.001 ^‡^
uK mEq/L	60–231	134.30 (62.50–230.40)	149.95 (85.0–200.40)	53.50 (19.0–169.30)	<0.001 ^‡^
uK/uCrea	0.16–0.66	0.34 (0.15–0.64)	0.68 (0.22–1.30)	0.47 (0.16–0.76)	<0.001 *
FE K%	7.0–22.0	11.87 (6.20–22.79)	12.69 (8.63–27.12)	38.96 (7.91–128.76)	<0.001 ^‡^
uCa mg/dL	1.0–10.0	2.40 (1.0–11.60)	2.05 (1.10–3.50)	2.40 (0.80–9.40)	0.586
uCa/uCrea	0–0.03	0.01 (0–0.03)	0.01 (0–0.03)	0.02 (0–0.14)	<0.001 ^‡^
FE Ca%	0–0.26	0.09 (0.05–0.45)	0.10 (0.04–0.26)	0.72 (0.10–11.15)	<0.001 ^‡^
uP mg/dL	90.0–385.0	187.70 (105.20–381.30)	230.0 (103.50–377.30)	58.10 (9.7–268.20)	<0.001 ^‡^
uP/uCrea	0.25–1.13	0.51 (0–0.89)	1.16 (0.32–2.26)	0.55 (0.04–0.87)	<0.001 ^¶^
FE P%	0–40	16.51 (8.28–37.32)	17.20 (9.54–38.15)	31.55 (3.02–54.99)	0.002 ^‡^
uCl mEq/L	51–366	179.40 (85.0–330.0)	87.0 (10.50–133.0)	190.50 (56.0–386.0)	<0.001 ^‡^
uCl/uCrea	0.13–1.10	0.43 (0.15–1.08)	1.02 (0.14–2.47)	0.64 (0.09–1.29)	0.004 ^†^
FE Cl%	0.18–1.26	0.55 (0.20–1.08)	0.80 (0.26–1.97)	1.42 (0.21–9.15)	<0.001 ^‡^
uMg mg/dL	0.76–17	11.06 (3.0–19.40)	8.07(1.04–14.64)	4.15 (0.76–9.70)	0.001 ^‡^
uMg/uCrea	0–0.05	0.03 (0.01–0.06)	0.05 (0–0.09)	0.04 (0–0.11)	0.051 ^†^
FE Mg%	0.05–3.23	1.92 (0.41–3.22)	1.72 (0.21–3.87)	6.38 (0.15–31.61)	<0.001 ^‡^
Total bands number	–	13 (9–15)	17 (5–24)	15 (6–21)	0.016 ^∞^
HMM number	–	6 (5–7)	6 (2–8)	4 (1–9)	0.049 ^‡^
LMM number	–	7 (3–10)	11 (3–17)	9 (5–16)	0.024 ^†^

Data are reported as median and range (in brackets). FeMV = Feline morbillivirus; CKD = chronic kidney disease; RI = reference interval; USG = urine specific gravity; uCreatinine = urinary creatinine; uUrea = urinary urea; uTP = urinary total proteins; UPC = urine protein:creatinine ratio; uGlucose = urinary glucose; uNa = urinary sodium; FE Na = fractional excretion of sodium; uK = urinary potassium; FE K = fractional excretion of potassium; uCa = urinary calcium; FE Ca = fractional excretion of calcium; uP = urinary phosphorus; FE P = fractional excretion of phosphorus; uCl = urinary chloride; FE Cl = fractional excretion of chloride; uMg = urinary magnesium; FE Mg = fractional excretion of magnesium; HMM = high molecular mass protein; LMM = low molecular mass protein; * Statistically significant difference among the three study groups; ^†^ Statistically significant difference in healthy compared with CKD and FeMV cats; ^‡^ Statistically significant difference in CKD compared with healthy and FeMV cats; ^¶^ Statistically significant difference in FeMV compared with CKD and healthy cats; ^∞^ Statistically significant difference between FeMV and healthy cats.
